# Current Insights on Treatment Adherence in Prevalent Dermatological Conditions and Strategies To Optimize Adherence Rates

**DOI:** 10.7759/cureus.69764

**Published:** 2024-09-19

**Authors:** Nicoleta Cîrstea, Ada Radu, Cosmin Vesa, Andrei Flavius Radu, Alexa Florina Bungau, Delia Mirela Tit, Carmen Delia Nistor Cseppento, Alexandra Georgiana Tarce, Simona Gabriela Bungau

**Affiliations:** 1 Dermatology, Faculty of Medicine and Pharmacy, Doctoral School of Biomedical Sciences, University of Oradea, Oradea, ROU; 2 Pharmacology, Faculty of Medicine and Pharmacy, Doctoral School of Biomedical Sciences, University of Oradea, Oradea, ROU; 3 Diabetes and Endocrinology, Faculty of Medicine and Pharmacy, Doctoral School of Biomedical Sciences, University of Oradea, Oradea, ROU; 4 Preclinical Disciplines, Faculty of Medicine and Pharmacy, Doctoral School of Biomedical Sciences, University of Oradea, Oradea, ROU; 5 Pharmacy, Faculty of Medicine and Pharmacy, Doctoral School of Biomedical Sciences, University of Oradea, Oradea, ROU; 6 Psycho-Neuroscience and Rehabilitation, Faculty of Medicine and Pharmacy, Doctoral School of Biomedical Sciences, University of Oradea, Oradea, ROU; 7 Internal Medicine, Faculty of Medicine and Pharmacy, University of Oradea, Oradea, ROU

**Keywords:** acne, adherence, dermatology, pharmacists, psoriasis, treatment

## Abstract

Adherence to prescribed medication regimens is crucial for treatment efficacy and patient safety, but it remains a challenge in the medical field, particularly in dermatology, where adherence to prescribed treatments is being intensively evaluated and improved. This narrative review provides a comprehensive overview of adherence behaviors in dermatological diseases, including fungal skin infections, psoriasis, acne, atopic dermatitis, and chronic urticaria, aiming to update scientific information on adherence patterns and management strategies in these highly prevalent conditions. Furthermore, the importance of a holistic approach that integrates patient-centered and physician-centered strategies to optimize treatment outcomes and enhance adherence in dermatological care is highlighted. The role of technological advancements in promoting adherence is also discussed, with an emphasis on the potential for digital solutions to facilitate medication management. Future perspectives underscore the need for targeted interventions to address the multifaceted barriers to adherence, including treatment complexity, healthcare accessibility, and patient-provider communication. By addressing these challenges, healthcare providers can enhance patient satisfaction, improve therapeutic outcomes, and mitigate the adverse consequences of non-adherence in dermatological practice.

## Introduction and background

Adherence, defined as the way a patient follows the healthcare provider's prescribed medication regimen, plays a pivotal role in treatment efficacy and patient safety on a global scale [1,2]. In a recent comprehensive analysis, it was highlighted that failure to adhere to medication poses a significant financial strain on the healthcare system [3]. Moreover, according to World Health Organization research, only up to 50% of patients adhere to chronic treatment regimens [4].

Distinguishing between adherence failure and treatment inefficacy is crucial, given that non-adherence ranks as a primary cause of therapy failure [5]. The study of patient adherence presents substantial challenges within health behavior research. The scientific community argues that these challenges stem from various factors, including fragmented research structures, inadequate qualitative methodologies, overlooking patient perspectives, and the absence of integrative frameworks [6].

Recent discourse among academics and clinicians emphasizes the importance of delineating adherence into three distinct stages to better understand adherence patterns and requirements. Initiation is the phase in which the patient begins treatment as prescribed after mutual agreement between patient and physician, facilitated through collaborative decision-making processes. It marks the commencement of the therapeutic journey and sets the foundation for subsequent adherence behaviors. In the implementation phase, adherence extends to meticulous compliance with prescribed guidelines, including dosage, frequency, timing, and other administration considerations. This phase requires patients to diligently follow the prescribed regimen to maximize treatment effectiveness. Finally, the persistence phase refers to sustaining therapy for the agreed-upon duration, which is essential for achieving optimal treatment outcomes. This phase quantifies the patient's ability to adhere to the treatment regimen over time, often measured using methods such as Kaplan-Meier 'survival analysis'. Persistence reflects the patient's commitment to continuing treatment despite potential challenges or obstacles [7].

Non-adherence to long-term health prescriptions is associated with increased healthcare resource utilization, exacerbation of symptoms, and even mortality [8,9]. Employing a patient-centered strategy could assist healthcare providers in identifying possible factors contributing to non-adherence. To narrow the disparities in practice, it is essential to acknowledge the barriers faced by patients. Patients may inadvertently or deliberately neglect their medication regimen. Inadvertent non-adherence may stem from lapses in memory, intricate treatment plans, apprehension of adverse reactions, patient convictions, or mental health conditions [10,11].

While patients are often deemed responsible for subpar adherence, there exists ample opportunity for physicians and the healthcare system to bolster adherence behavior. Providers may inadvertently exacerbate patients' adherence challenges by prescribing costly medications beyond their financial means, suggesting convoluted regimens that are hard to follow, and inadequately educating patients about medication advantages and drawbacks. These circumstances culminate in a strained patient-provider dynamic, further jeopardizing adherence [12].

Elaborate treatment protocols frequently confuse patients and dampen their motivation, resulting in suboptimal adherence. Initiating numerous medications during a single office consultation or augmenting prescriptions atop an extensive medication regimen can diminish patient adherence. Streamlining treatment protocols alleviates the treatment load and enhances the probability of adherence. Patients are more inclined to comply with once-daily regimens as opposed to those requiring twice-daily administration. Consolidating medications similarly alleviates the treatment burden and enhances adherence. Inadequate communication from healthcare providers to patients can also precipitate adherence issues [13-15].

Obstacles within the healthcare system that impede adherence include constrained healthcare accessibility, limited formularies, transitions to alternative formularies, and exorbitant medication expenses, copayments, or both [16-18]. The repercussions of inadequate adherence manifest in adverse consequences, including heightened morbidity and mortality rates and increased costs for healthcare systems. Such outcomes have been consistently evidenced across various chronic ailments, including but not limited to tuberculosis, rheumatic disorders, mental disorders, diabetes, cardiovascular disorders, HIV, and asthma [19].

Despite the common belief that adherence is subpar for asymptomatic conditions such as hypertension, research indicates that adherence is also lacking for dermatological conditions that visibly impact patients' daily lives. Moreover, dermatology, in particular, grapples with the detrimental effects of non-adherence, with half of individuals with chronic skin conditions failing to adhere to prescribed treatments [20,21].

Various therapeutic modalities are available for individuals with dermatological conditions, encompassing systemic interventions (administered orally or via injection), phototherapy, and topical treatment [3]. Although adherence to systemic therapies falls short of optimal levels, adherence to topical treatments, frequently recommended in dermatology, is notably deficient [22]. Moreover, the chronic nature of these conditions and the often-multifaceted treatment strategies exacerbate challenges in medication adherence. Inadequate adherence frequently culminates in unfavorable outcomes, necessitating the prescription of more potentially dangerous and expensive medications [23].

Various patient-related factors, including socioeconomic status, health literacy, age, and ethnicity, influence adherence to oral medications. Additionally, treatment complexity and duration significantly impact adherence behaviors. Similarly, adherence to topical treatments is affected by comparable factors, with evidence suggesting that educational interventions and simplified dosing regimens can enhance adherence rates [24]. Consistent application of topical treatments is crucial for patients with skin diseases to experience therapeutic improvements [25]. Enhanced treatment adherence not only contributes to patient satisfaction but also improves overall therapeutic outcomes synergistically [26].

This narrative review aims to provide a comprehensive overview of adherence to treatment regimens in highly prevalent dermatological diseases, including fungal skin infections, psoriasis, acne, atopic dermatitis, and chronic urticaria. By synthesizing current literature and introducing updated information on adherence patterns and management strategies, this review seeks to contribute to the scientific understanding of adherence behaviors in dermatological conditions and offer insights into improving treatment outcomes and the role of pharmacists in managing adherence to treatment. Furthermore, it provides a synopsis of the technological advancements that may promote adherence.

## Review

Adherence to antifungal therapy and strategies to improve it

It is estimated that one to two billion individuals worldwide suffer from fungal infections of the skin, nails, and hair. Moreover, mucosal Candida infections impact tens of millions of people, while severe fungal infections affect 150 million individuals [27]. According to estimates, the number of fatalities from major fungal infections is comparable to that of tuberculosis [28,29]. Fungal skin infections rank among the most prevalent ailments, presenting either as primary conditions or resulting from systemic infection dissemination [30].

Currently, there are five prevalent categories of antifungal medications, including polyenes, pyrimidine analogs, azoles, echinocandins, and allylamines. These drugs serve as treatment options for both superficial and systemic fungal infections [31].

Treatment success may be hindered by antifungal resistance, emphasizing the importance of antifungal susceptibility testing in guiding therapy [32]. An emerging clinical challenge in antifungal therapy is the development of resistance among fungal pathogens to existing drugs. Numerous factors, including those related to the host, the fungus, and the environment, influence this resistance development. Antifungal resistance arises due to a combination of sexual reproduction, adaptive phenotypic plasticity, mutations in target genes followed by selective pressure, horizontal gene transfer, and chromosomal aneuploidy [31,33]. Additionally, certain novel immunomodulatory medications may increase the risk of mucocutaneous candidiasis in patients [34].

In general, adherence to treatment is optimized by simpler and shorter regimens. However, despite recommendations for once-daily dosing to enhance adherence, certain antifungal medications, such as miconazole, sertaconazole, and clotrimazole, still require twice-daily administration. Treatment efficacy in mycotic infections is influenced by various factors, including skin type, lesion characteristics, and patient-specific needs. Yet, therapeutic success is heavily reliant on patient adherence, making it essential to consider the patient's likelihood of complying with the treatment plan. Patient compliance with dermatomycosis therapy tends to decline with prolonged treatment duration and increased daily applications, particularly after symptom resolution [35].

Improving adherence often involves enhancing communication between healthcare providers and patients, bolstering patient education, and simplifying dosing schedules. Isolated interventions to improve adherence are generally deemed ineffective, with effective oral communication during physician-patient interactions being crucial. Clinicians must tailor medication choices to accommodate patients' lifestyles, while therapeutic regimens should be designed with realistic expectations of patient behavior, aiming for once-daily dosing and shorter treatment durations whenever feasible [35,36].

Adherence in psoriasis and strategies to improve it

Psoriasis is an autoimmune disease that can occur in many different clinical forms, such as flexural, pustular, guttate, erythrodermic, or plaque [37]. It is primarily caused by immune system-mediated inflammation and has a significant genetic component. Red, flaky patches on the skin or in the joints are the hallmarks of psoriasis, a chronic inflammatory skin or joint condition [38].

Psoriasis affects up to 3% of the global population [39]. Furthermore, approximately 60% of patients with psoriasis believe that their quality of life (QoL) is significantly impacted by their condition, regardless of its severity [40]. While psoriasis may not pose an immediate threat to life, it decreases QoL to a degree comparable to other serious medical conditions [41].

The type and severity of psoriasis should determine how it is managed. Treatment options for psoriasis include topical corticosteroids (TCS), phototherapy, oral systemic drugs like methotrexate (MTX) and cyclosporine, biologic agents, and topical steroid-sparing medications such as vitamin D analogs, retinoids, and tacrolimus. For complex cases, a multimodal therapy strategy may be required. However, adherence to treatment can be hampered by the complexity of these regimens [42].

Compared to other chronic dermatological conditions, psoriasis patients exhibit the lowest primary adherence rate, with 44% of individuals neglecting to take their prescribed medication [9]. In a study evaluating adherence to oral and topical psoriasis treatments, self-reported adherence rates by patient interview were 92.0%. However, mean medication adherence was only 60.6% when measured by medication weight and pill count [43].

According to self-reported questionnaire data, adherence rates stood at 75% for topical treatments, 93% for phototherapy, 96% for oral medications, and 100% for biologics [44]. There is a stronger correlation between biologic therapy and improved clinical outcomes. Physician suggestions, patient preferences, and other aspects of therapy satisfaction are directly related [45].

The percentages obtained for adherence among psoriasis patients to different therapies are influenced by numerous factors (i.e., assessment modalities, types of therapies, modes of administration, etc.), with results from various scientific studies differing greatly. The lowest rates of adherence are linked to topical therapies [46].

Results from medical evaluations showed that the percentage of psoriasis patients who consistently applied their topical medication as prescribed varied between 39% and 72% [47]. Furthermore, the rate of adherence to TCS in psoriasis patients was around 30-50% [48].

Although 77% of polled patients reported non-adherence, topical therapies had the greatest prevalence of non-adherence (97%), with the most common reason cited being a lack of treatment efficacy [46]. According to a systematic evaluation, patients adhering to topical psoriasis therapy observed between 50% and 60% of the anticipated frequency of applications. Additionally, patients applied varied doses, ranging from 35% to 72% of the recommended amount [49]. Long-term adherence rates were even lower compared to short-term rates: after eight weeks, only 51% of participants continued to use their topical psoriasis treatments, down from 84.6% at the beginning [50].

Comparable low adherence rates have been found for several topical treatments: salicylic acid compounds account for 41%, vitamin D derivatives for 57%, and topical steroids for 50% of cases [49]. Furthermore, poor adherence to topical therapies is frequently attributed to a number of factors, including ineffectiveness, time investment during application, and unsatisfactory esthetic qualities of the particular preparation. A patient study conducted on 1,291 individuals with psoriasis across Europe found that reasons for non-adherence included esthetic and physico-chemical issues with the pharmaceutical form (29%), limited efficacy (27%), more time commitment (26%), and adverse reactions (15%) [51]. Factors like staining of clothing (27%), staining of bedding (34%), the need for more frequent applications (41%), and inefficient absorption (44%) were frequently cited as causes of non-adherence [52].

A cross-sectional questionnaire study aimed to explore the perspectives of dermatologists and patients with psoriasis regarding adherence to topical treatments. Psoriasis specialists collaborated to design the survey, which was completed by 50 patients and 26 dermatologists. More than 50% of dermatologists questioned patients about adherence approximately 20% of the time, according to the physician survey. It was widely believed that patients had lofty expectations for the results of their treatments. According to nearly 40% of dermatologists, more than 60% of patients followed the recommended topical treatment regimen. Moreover, 15% of patients in the survey reported they were not given sufficient information regarding the medication. The physical qualities of the products were also a source of complaint for roughly 20% of patients. Most patients were optimistic about the efficacy of available topical therapies and anticipated noticeable results in as little as two to four weeks. Forgetfulness was the most common reason for decreased adherence, while concerns about adverse effects and inconvenience often led to the discontinuation of topical treatments [53].

Systemic therapy for moderate-to-severe psoriasis is often required for an extended period. A retrospective, comparative cohort study examined the adherence of new users of adalimumab, etanercept, ustekinumab, acitretin, and MTX. Adherence to etanercept, adalimumab, and ustekinumab was higher, while adherence to acitretin was lower among the 22,472 new users of systemic medicines, compared to MTX. These findings aligned with earlier research [54,55], demonstrating higher adherence to biologics than to other systemic treatments [56].

However, during a retrospective examination of 2,707 patients who commenced ustekinumab (10.3%), infliximab (11.7%), adalimumab (40%), and etanercept (37.9%), it was found that 38% of individuals exhibited adherence, with 46% ceasing treatment over a 12-month follow-up period, showing poor adherence [57].

In another study of 2,130 patients who received biologic treatments, 447 (or 21%) had access to procedures according to disease severity. According to the medication possession ratio (MPR) and the proportion of days covered, the average adherence rate was significantly lower than 75%. In total, 52.2% had a mean MPR greater than 80%, with an average of 297.6 days of biologic persistence. Disease severity was inversely related to persistence and adherence, with patients exhibiting mild psoriasis showing higher adherence and persistence than those with moderate and severe psoriasis [58].

Through multiple rounds of patient assessments, key themes regarding the causes of non-adherence in psoriasis were identified. Patients' ability to participate in social events and manage their work responsibilities was frequently hindered by their perception of psoriasis and its management as a burden and stigma within society. The belief that psoriasis is a chronic, lifelong illness with poor symptom management and uncertain response to treatment contributed to psychological stress, frustration, and pessimism, making adherence more difficult. Moreover, patients frequently felt that medical professionals lacked empathy and rarely addressed the challenges associated with using medications [59].

The high cost of therapy, lack of information, perceptions of treatment effectiveness, ineffective communication, forgetfulness, and a lack of accountability are some of the obstacles affecting psoriasis adherence. Key elements needed to increase adherence include fostering a compassionate, supportive environment and making the patient responsible for ensuring their own adherence. Several strategies can help establish accountability and compassion, such as rescheduling clinic visits for an early return after prescribing medication, requesting that patients report to the physician virtually, assigning follow-up tasks to other healthcare team members, building strong rapport between doctor and patient, educating patients and encouraging self-management, and addressing treatment burden and non-persistence [7].

During consultations, doctors and other healthcare professionals can learn about the patient's daily activities and how their skin condition affects them [60]. Physicians can assist patients with financial difficulties by prescribing affordable medications, helping them adhere to their primary care regimen [61]. Greater emphasis should be placed on one-on-one meetings between patients and healthcare professionals, as well as ensuring convenient access to the healthcare system, rather than relying on reminder calls and educational materials [62]. Patients who are struggling to manage their psoriasis may benefit from more frequent follow-up appointments to improve adherence [63].

Patients may be more likely to take their medication as prescribed if it is recommended by nurses, as patients often view nurses as more attentive listeners and knowledgeable about medication compared to doctors [64]. Moreover, from the patient's perspective, adherence may improve if there is less administrative burden, appointments are available when needed, and communication with the hospital is consistent [65]. Improving short-term treatment outcomes may be possible with customized training on the proper administration of topical medications [66].

**Figure 1:**
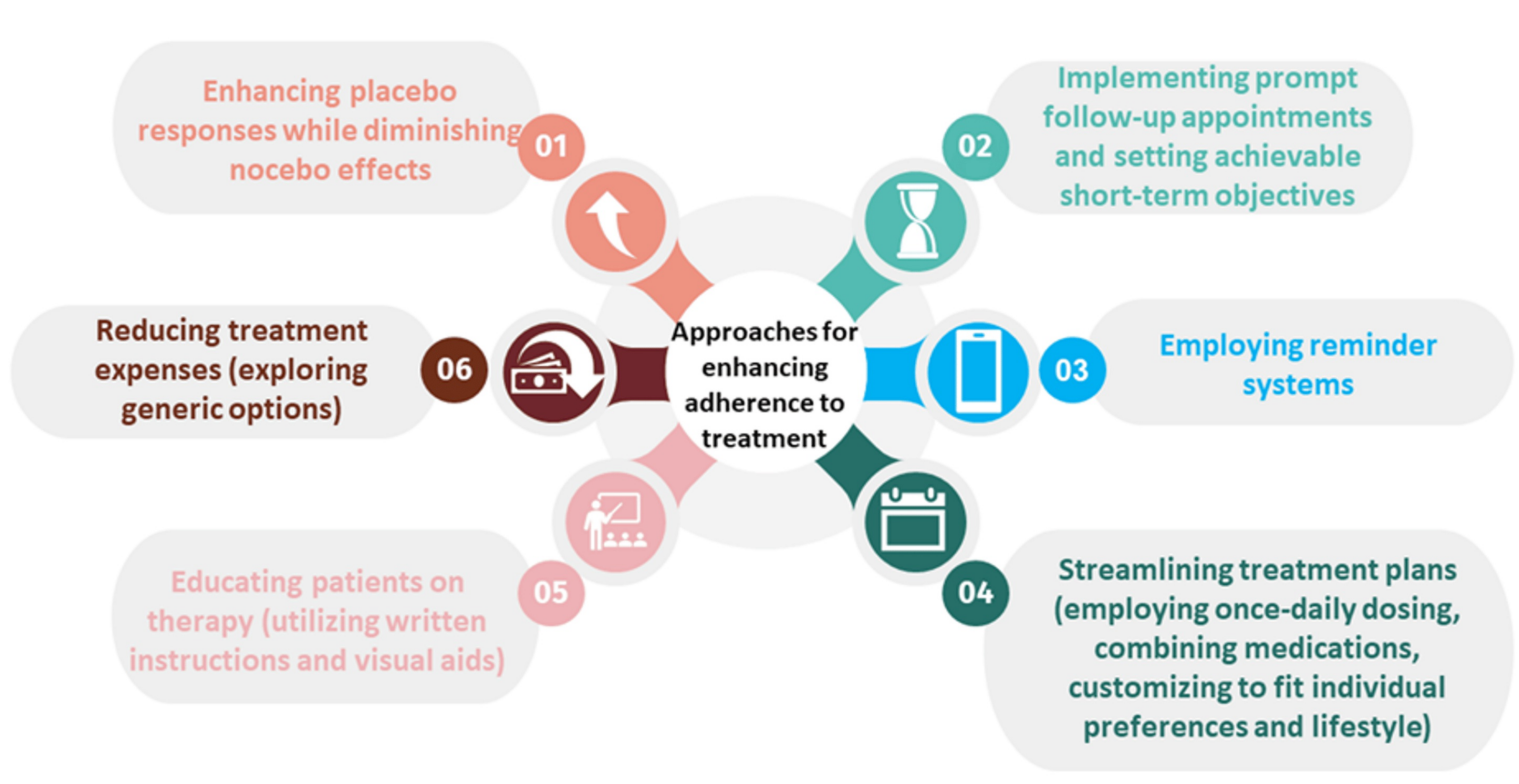
Methods to improve treatment adherence. Credits: Andrei Flavius Radu.

Empowering patients may be achieved through a patient-centered strategy [62]. Figure 1 shows relevant approaches that can be implemented to increase adherence to treatment [5].

It is essential for healthcare professionals to involve patients in making choices and to show empathy and compassion for their irritability and frustration with the illness, as patients frequently endure embarrassment and life-altering manifestations [60].

Adherence in atopic dermatitis (AD) and strategies to improve it

AD is a long-term inflammatory skin condition that usually appears in early childhood and lasts into adulthood [67]. Moreover, up to 20% of children globally are affected by AD [68,69]. AD is characterized by pruritic, eczematous skin, exhibiting a pattern of symptoms that alternate between periods of remission and relapse [70].

Numerous topical treatments are used in management, including emollients, topical calcineurin inhibitors (TCIs), and TCS. In clinical practice, phototherapy and systemic treatments are warranted when topical therapies fail to yield desired outcomes. The primary anti-inflammatory medications used to treat AD are tacrolimus ointment and TCS [70]. The introduction of dupilumab, which targets the interleukin-4 receptor α and interleukin-13 and is administered subcutaneously every two weeks to individuals aged 12 and older with moderate-to-severe AD not well managed by topical prescription medications or for whom those therapies are not recommended, has altered the AD therapy model in the past four years [71].

The therapeutic landscape for AD has recently expanded with the approval of Janus kinase (JAK) inhibitors such as upadacitinib, abrocitinib, and baricitinib, which complement biologics like dupilumab, nemolizumab, and tralokinumab [72]. According to updated European guidelines, these JAK inhibitors are now incorporated into the six systemic therapies recommended for adults with severe forms of AD [73].

Among the latest topical JAK inhibitors are ruxolitinib (JAK1/2) and delgocitinib (pan-JAK). Ruxolitinib cream has successfully met both primary and secondary endpoints in phase 3 trials for patients with mild-to-moderate AD, demonstrating a favorable safety profile with minimal treatment-emergent adverse events (TEAEs). In Japan, delgocitinib ointment has been approved for the treatment of both pediatric and adult AD cases. Oral formulations of JAK inhibitors, specifically baricitinib (JAK1/2), abrocitinib (JAK1), and upadacitinib (JAK1), have shown efficacy by achieving primary and secondary endpoints across multiple clinical studies focusing on moderate-to-severe AD. The adverse events most commonly reported with these oral agents were generally mild to moderate and included headache, nausea, acne, upper respiratory tract infections, and occasional herpes infections and specific laboratory abnormalities. Overall, JAK inhibitors represent a promising class of targeted therapies, potentially revolutionizing the management of AD [74].

Studies on the variables influencing patients' adherence to therapy have the potential to significantly enhance patient outcomes rapidly and inexpensively. Patients may be nonadherent in two distinct ways: either they neglect to take their medication as prescribed (i.e., secondary nonadherence), or they do not redeem their prescriptions (i.e., primary nonadherence) [75].

Therapy misunderstanding and the chronicity of the disease are additional reasons for poor adherence, along with the inconvenient nature of treatments, forgetfulness, and fear of side effects, especially concerning TCS. It is essential to investigate novel approaches that boost patients' confidence in their abilities and adherence to treatment plans to lessen the financial burden of AD while simultaneously improving patients' treatment outcomes, QoL, and overall health [76].

Adherence rates in AD have been evaluated in numerous studies. Due to the high treatment expense, inconvenience, and steroid phobia, nonadherence is common among AD populations. In an outpatient dermatology clinic, 322 participants' primary nonadherence was evaluated by examining the number of medications filled within four weeks of the consultation. A total of 31% of the 137 AD participants who received new prescriptions failed to fill them [77].

Many individuals continue to fail to take their medication as prescribed, even after filling their prescriptions. In another study, the average delay in therapy was seven days, and only 50% of patients started medication during an acute AD flare [6]. Numerous patients neglect to take the recommended dosage of medication for the long-term management of AD. Medication misuse or underuse by patients might result in an inadequate response to treatment. In a study assessing the effectiveness of topical tacrolimus in adults with AD, 20.9% of individuals used the recommended dosage, 12.4% overused it, and 66.7% underused it [78].

Even though patients self-report excellent levels of adherence, the electronic monitoring system's adherence rate is not ideal. Moreover, 70% of the 25 AD patients who were taking hydrocortisone 17-butyrate 0.1% twice a day in one of three vehicles did so as prescribed, even though almost all patients claimed to be using the drug correctly [79].

Even short-term therapy is associated with low adherence. The mean adherence rate, as determined by electronic monitoring, was 40% among 10 participants with mild-to-moderate AD who received instructions to use fluocinonide cream 0.1% twice daily for 5 days [80].

The first-line treatment for AD is TCS, and most evidence on adherence is derived from the use of these topical medicines. However, the recommended course of care for treating and preventing AD involves using moisturizers daily [81]. Emollients, humectants, and occlusive agents are examples of moisturizers. If these agents are inconvenient for the patient and do not fit their preferences, nonadherence could become a concern. The three most often mentioned reasons for not using moisturizers are expense (23%), skin discomfort (27%), and the time-consuming application process (22%) [82].

In another study, the effect of an online survey on the adherence of 28 patients with moderate AD to topical crisaborole (i.e., topical phosphodiesterase 4 inhibitor) 2% ointment was evaluated. After six weeks of weekly questionnaires, the intervention group switched to monthly surveys for the next twelve months to gauge adherence. Furthermore, 60% of the patients who remained in the trial showed adherence. In adults, 49% of the control group and 45% of the intervention group were adherent. Both the control and treatment groups of children had low adherence rates: 27% and 29%, respectively. Overall, adherence to medication was poor, and participation in the survey did not lead to better adherence. However, compared to the adult control group, a higher percentage of intervention group members completed the study. Feeling supported and maintaining therapy may be easier for patients if they receive regular communication from their provider [83].

Second-line treatments, such as TCIs, are suitable for thin, sensitive skin types that might not be able to tolerate TCS. TCIs approved for AD include pimecrolimus cream and tacrolimus ointment. In a study evaluating adherence to topical tacrolimus application instructions, which involved 200 AD patients, the mean daily application count was 1.75 ± 0.53, and adherence rates sharply decreased from the first to the second week (73.5% vs. 61%).

Adherence in AD is impacted by several challenges, including inadequate education, preconceived notions, discomfort, a poor patient-physician relationship, cognitive impairment, a lack of accountability, and expense [7].

Patients frequently worry about the potential risks of TCS and may not be fully aware of the benefits of moisturizers and additional medications like TCI in treating AD. In a poll of caregivers whose children had dermatological issues, the most frequently mentioned concern regarding medication was the possibility of negative side effects [84]. Two typical obstacles to adherence are inadequate communication and a misalignment with the preferences of the patients. Patients may experience confusion over the various TCS potencies and the proper administration of the drug. Moreover, 18.2% of respondents to a poll on the causes of nonadherence to topical medicines cited confusing instructions as a common impediment to optimal care [53]. Incorporating necessary medications into a regular daily regimen may assist with forgetfulness. One strategy could be to take the medication each morning after breakfast [85].

Interventions such as instructional workshops, written strategies, reminder devices, early follow-up visits, and switching to less expensive generic prescriptions may help increase adherence and overcome barriers that lead to nonadherence. Educational workshops can be particularly useful by providing practical training in medication administration, addressing concerns about side effects, and helping patients and caregivers become more knowledgeable about the illness and available treatments. Personalized, in-person training during these sessions assists patients and caregivers in managing AD [7,48].

Patients with AD can improve their condition by participating in several informative courses. In a research study, 204 AD participants were randomized to receive standard care or six two-hour instructional seminars. Using an interdisciplinary approach, pediatricians, nutritionists, and psychologists participated in the sessions. At a one-year follow-up, 82% of the intervention group and 67% of the control group were using regular skin care products, including TCS and emollients. Approximately 65% of the intervention group and 38% of the control group used TCS. Additionally, during the 1-year follow-up, the intervention group had lower treatment costs than the control group, indicating improved self-management and reduced healthcare utilization [86].

Eczema Action Plans (EAPs), also known as written action plans, are useful tools that incorporate patients' preferences and provide precise instructions. Many AD patients and physicians find EAPs helpful for enhancing self-management. According to 79% of pediatric dermatologists, EAPs are recommended as a way to increase adherence [87]. A self-reported survey of 35 AD caregivers found that 86% believed EAPs helped manage AD flare-ups, and 68% linked EAPs to an improvement in the condition [88].

Reminder texts have emerged as a cutting-edge method for increasing adherence. A 6-week pilot study evaluated the impact of daily text message reminders on 25 AD patients' adherence. Participants completed a questionnaire about how often they forgot to take their medication and kept a self-reported medication diary. By week 6, 72% of participants showed improved adherence compared to baseline. Furthermore, 88% found the text reminders helpful, and 84% said they would continue using them. In another six-week pilot project, researchers selected adults with moderate-to-severe AD, using a smartphone application to disseminate information on symptoms and triggers, set treatment reminders, provide lifestyle advice, and offer overall support. The intervention showed an average improvement in clinical symptoms of 44%, patient-reported global severity improvement of 46%, and QoL enhancement of 41%. Improvements in skincare, disease-related information, and avoidance of triggers were also noted from baseline to post-intervention. Greater clinical gains were seen in patients who adhered more closely to their treatment plans, suggesting a strong interaction between the two variables [76].

White coat compliance, where medication adherence increases around the time of follow-up appointments, can also play a role in improving adherence. Increasing the number of promptly scheduled and frequent follow-up visits after initiating a new AD treatment regimen enhances the likelihood of correct medication administration and prescription redemption. Since AD patients are prone to sticking with their medication once they start to feel better, this can result in an earlier treatment response and higher long-term adherence. In a randomized controlled trial of 30 AD patients using tacrolimus ointment 0.03% twice daily, an additional office visit led to better adherence compared to the control group [89].

A clinical study involving 1,739 subjects showed that AD patients satisfied with their healthcare providers' connection, the information provided, or explanations about AD had higher adherence scores than those with lower health literacy skills. Unmet medical needs for AD included medical treatment expenses, hospital visit access, and explanations about illness prognosis [90].

One significant and often overlooked factor contributing to nonadherence is the high cost of AD treatments. Using electronic medical records to substitute the most expensive original medication with a more affordable generic topical steroid is one potential approach [84,91].

Adherence in acne and strategies to improve it

Bacterial colonization, abnormal keratinization, increased sebum secretion triggered by androgenic hormones, and inflammation all contribute to the common skin disorder known as acne vulgaris [92,93]. Over 85% of teenagers suffer from acne [94], and the condition can worsen in adulthood. Although acne is not life-threatening, it is associated with lower self-esteem and reduced QoL [95].

The clinical course and severity of acne can vary greatly, often requiring long-term treatment. Numerous medications are available, ranging from topical retinoids and antibiotics to systemic therapies like oral antibiotics and hormone-related substances [96-97]. For mild to moderate acne, topical treatments are the first line of defense, while severe cases may require oral antibiotics or isotretinoin [98].

Adherence to basic acne treatment regimens tends to be suboptimal, and adherence to more complex regimens is notably poorer. Acne patients are prone to primary nonadherence, meaning they fail to procure or administer a prescribed drug. Within the first three months, 27% of acne prescriptions were not filled. By three months, 34% of prescriptions remained unfilled, though only 6% of patients cited nonadherence as the primary reason for this [99].

Missing doses, stopping medication early, or misusing prescriptions are examples of secondary nonadherence. In a study of 428 people with acne, 76% reported poor adherence. Fifty-two percent of patients using topical monotherapy and 49% of those using combination therapy showed poor adherence. Good adherence was more common among patients who understood acne and how to manage it [100].

In a retrospective cohort study of 24,438 Medicaid users' adherence to acne medication, only 12% were adherent. Age, gender, frequency of refills, and the number of drug classes used were significant factors in adherence [101]. Furthermore, just 4% of children and 13% of teenagers with acne covered by Medicaid adhered to their medications. Overuse, drug holidays, and confusion about dosage instructions were additional issues contributing to treatment failure. Adherence can also decline over time [102].

Oral therapy, including contraceptives (49%), antibiotics (4%), glucocorticoids (2%), and retinoids (57%), demonstrated better adherence compared to topical treatments in an adherence evaluation [103].

First-generation all-trans retinoic acid (e.g., tretinoin) and third-generation topical retinoids (e.g., adapalene, tazarotene) are key treatments for comedonal and inflammatory acne [104]. Retinoids are frequently stopped prematurely due to initial irritation, delayed improvement, and complex treatment plans [52]. Topical therapies can be time-consuming and require prolonged use to see results, contributing to poor adherence [105].

Topical antibiotics like dapsone, erythromycin, and clindamycin are also commonly prescribed. These antibiotics target Cutibacterium acnes and Staphylococcus aureus [104]. However, they should not be used as monotherapy due to the risk of antibiotic resistance. Benzoyl peroxide is often prescribed alongside them to mitigate this risk. Only 45% of patients use topical antibiotics daily, with unintentional nonadherence often due to misinformation or forgetfulness. Some patients stop using their medication once they believe their condition has improved [106].

Sixty percent of patients rated their topical acne treatment adherence as acceptable or excellent in a 2010 broad-scale global study [107]. The duration between applying the medication and seeing results is a major deterrent for many people who use topical treatments. Many people take medication with the expectation that it will instantly improve their condition. It could be challenging for teenagers to wait longer for positive results [108].

Systemic therapy is often required to manage acne, as topical treatments alone may not be sufficient [109]. Oral medications generally lead to higher adherence rates than topical treatments [110]. Isotretinoin, in particular, had a higher adherence rate compared to other systemic treatments like hormonal medications and antibiotics. An adherence rate of 87.5% was reported for the first course of isotretinoin, dropping to 60.5% for subsequent courses [103].

Participants in a tertiary dermatological clinic-based, non-randomized interventional study were compared to a control group that was age- and treatment-matched. After seeing a 10-minute video about acne, patients in the control group were counseled on how to control their condition. Both concrete measures, such as tube weights and pill counts, and subjective measures, such as the ECOB (i.e., Elaboration d'un outil d'evaluation de l'observance des traitements medicamenteux) questionnaire, were used to ascertain the adherence rate. Acne severity was evaluated with the use of the Global Acne Grading System and the Comprehensive Acne Severity Scale. Moreover, 100 patients finished the 12-week trial, and at week 12, adherence to 5% benzoyl peroxide gel was 71% vs. 57.9% at baseline, and adherence to 0.05% tretinoin cream was 58.7% vs. 45.4% at baseline. Yet, there was no improvement in oral medicine adherence because of the intervention program. There was a statistically significant increase in the proportion of patients whose disease severity improved after intervention (47.3% vs. 39.1%). Forgetting to take their medication was listed as the leading cause of treatment non-adherence by 54% of sufferers, followed by a hectic lifestyle by 41% and a lack of understanding of acne by 26% [111].

Patients' adherence to their acne medications was positively correlated with their QoL [52]. Increasing age, female gender, and having a job are all weaker indicators of adherence. Not having enough time to take the medication as prescribed was the most often cited cause of nonadherence. Patients with acne who took their medicine as prescribed had better health outcomes, and those whose prescriptions required less frequent dosage were the most adherent. Adherence can be reduced when there is more time between visits to the specialist [98].

Lack of knowledge, heightened sensitivity to side effects, complicated treatment regimens, low patient satisfaction, high treatment costs, and a hectic lifestyle are all obstacles to patient adherence [112,113].

Acne patients, particularly teenagers, often have hectic schedules. Skincare regimens that require significant time are not appealing. Teenagers prefer a straightforward morning routine, and adherence to multiple-dosing regimens can be challenging due to extracurricular activities. Patients may hesitate to take their medication in front of peers and may occasionally forget. A potential solution could be to apply combination therapy every night before bed [108]. This reduces the number of medications the patient needs to purchase, store, and apply, as well as the number of steps involved. Adherence improves when the treatment minimally interferes with the patient’s daily activities [103].

A crucial element of adherence is patient awareness [114]. Establishing reasonable expectations is a key part of patient education. Teens are accustomed to seeing immediate results and may become frustrated, mistaking slow improvement for treatment failure [108]. Additionally, the myth that effective treatment is unattainable is reinforced by previous failures with over-the-counter supplements. Teenagers who receive active guidance and reassurance are more likely to adhere to prescribed treatment plans [100].

Clinicians and pharmacists might find it useful to provide patients with a written plan in addition to explaining the course of therapy. In one randomized study involving 80 patients, 80% adhered to both oral and written counseling, compared to only 62% in the control group. Within 15 days of starting treatment, the written counseling group also received a phone call with further instructions. Adherence was evaluated through self-reporting at 30, 60, 90, and 6 months. The study found that written counseling increased adherence during the first month of treatment [115].

Forgetfulness is a common barrier to adherence [116]. Patients can be reminded using various methods. In a meta-analysis, eight out of eleven trials demonstrated a statistically significant improvement in adherence among patients receiving reminders. Techniques such as interactive voice response systems, phone calls, text messages, video calls, programmed reminder devices, and pagers were used in these trials [117]. Web-based reminders are a viable strategy given teenagers’ familiarity with technology [118]. Although technology-based reminders are promising and cost-effective, more research is needed to determine whether texting and smartphone-based interventions enhance adherence [119].

Patients need to be determined to follow their medication regimen as prescribed for their therapy to be effective. Simple regimens are easier to integrate into daily routines, whereas complex, time-consuming therapies disrupt patients' habits. Asking patients to summarize their daily routines and preferences can be helpful. Some patients prefer taking medication before bed, while others may find the morning more convenient. Adherence improves when treatment plans account for these preferences [120].

There are several topical forms of acne medication, such as foams, creams, and gels. Offering patients a choice can improve adherence, as they may tolerate certain formulations better. For example, tazarotene 0.1% is available as a cream, foam, or gel. The foam formulation may enhance satisfaction and adherence in patients who find the tactile sensation of a gel or cream uncomfortable [121].

Through the white coat compliance phenomenon, more office visits could lead to better treatment outcomes [122]. The Hawthorne effect is the tendency for patients to behave differently when they are aware that they are being closely observed [123]. This explains why clinical studies frequently have better rates of adherence. Adherence rates increased around the time of office visits, according to observations made on 29 patients participating in a clinical study of psoriasis [122].

Sustaining adherence depends on patient satisfaction, and communication between the patient and physician is crucial [114]. Adherence can be improved by involving patients in the treatment process and reaching a consensus on the approach. Tailoring interventions to the needs of the patient or parent also yields better results. For instance, allowing patients to choose the type of formulation improves the tolerability of the therapy and, consequently, increases the likelihood that they will comply with the prescription [112].

Adherence in chronic urticaria and strategies to improve it

The presence of wheals and/or angioedema for a period of six weeks or longer is characterized as chronic spontaneous urticaria (CSU). Like chronic idiopathic urticaria, CSU is a painful disorder that can significantly diminish a person's QoL [124]. The global prevalence of CSU is estimated to range from 0.23% to 1.88%. There is a strong female predisposition, with women affected at twice the rate of men. Although all age groups are susceptible, the highest prevalence is found in individuals aged 40 to 60 [125,126].

A study on chronic urticaria aimed to examine patients' adherence to treatment and its impact on their QoL. The dermatology clinic at National University Hospital in Singapore recruited 103 participants for a cross-sectional study. A questionnaire was administered to assess compliance with treatment and overall well-being. Patients were ranked into high, medium, and low adherence using the Morisky 8-Item Medication Adherence Scale. The Chronic Urticaria QoL Questionnaire was used to evaluate QoL. Sleep disruption and itching received the highest median scores on the QoL scale. Additionally, 71.9% of patients were found to be nonadherent to their prescribed medication regimens. Differences in adherence were observed among patients who took their medication more frequently or only once daily. However, there were no significant differences in QoL between patients with poor and medium adherence [127].

Nonadherence to prescription drug regimens is a major issue in managing CSU. A study examined the Japanese translation of the Morisky Medication Adherence Scale-8 to assess adherence to urticaria treatment. The study included 3,096 registered dermatology patients who completed online questionnaires, along with 751 urticaria patients. A strong correlation was found between the number of hospital visits and adherence to oral medication. Age and prior knowledge of therapeutic efficacy influenced adherence to topical medications. The percentage of patients reporting symptom improvement varied across different dermatological conditions treated with oral medication. Dermatologists should be aware that adherence to urticaria treatment is relatively low. To improve adherence and achieve positive therapeutic outcomes, patients with urticaria should attend regular appointments and actively participate in their education [128].

Factors influencing treatment adherence

A multitude of factors targeting patients and providers lead to less-than-optimal health outcomes, and the healthcare system faces significant obstacles and financial strains as a result of medication nonadherence. The healthcare system includes patients, physicians, pharmacies, hospitals, insurance, and pharmaceutical firms [129].

Several factors influencing medication adherence, such as patient beliefs, fear of side effects, patient preferences, complicated treatment plans, pharmaceutical costs, and insurance issues, are considered "intentional" factors. Additionally, there are "unintentional" factors contributing to nonadherence, such as forgetfulness, mental health conditions, educational gaps, poor communication, prescription refill failures, limited access to healthcare, and poor patient-physician interactions [130].

Physicians, pharmacists, and patients can reach a consensus on a practical treatment plan by discussing and balancing the patient’s goals and preferences. For instance, even though injectable treatments may improve disease control, some individuals with severe psoriasis may prefer oral medications over injectable ones [131]. Furthermore, physicians who prescribe costly drugs that patients cannot afford, suggest complicated regimens that are difficult to follow, or fail to inform patients about the advantages and disadvantages of the medication may contribute to poor adherence [13].

Since prescription pharmaceuticals are often costly, many patients cannot afford their medications. As a result, patients may not pick up their prescriptions, may quit treatment early due to economic concerns, or may skip doses to stretch out the medication. Caretakers may not be aware of how much patients are paying for their treatment. Patients and providers frequently neglect to discuss costs before prescriptions are filled. Prescribing generic medications increases the likelihood that a patient will be able to afford their treatment [13,103].

Complicated treatment plans can cause patients to become confused and lose motivation, which lowers adherence. Prescribing multiple drugs after a single office visit or adding prescriptions to an already lengthy list can reduce patient adherence. Simplifying regimens can reduce treatment burden and improve patient compliance [13-15].

Patients are more likely to adhere to once-daily treatment schedules than to regimens requiring twice-daily administration. Combination drugs can also improve adherence and reduce the burden of therapy [13,14]. Inadequate adherence may also stem from healthcare professionals' failure to adequately communicate with patients. Providers may not fully explain the patient’s condition, the need for medication, the anticipated course of treatment, or potential side effects. Educating patients is an essential part of the clinical interaction, offering an opportunity to address patient concerns and build a strong patient-provider relationship. When healthcare professionals fail to provide basic information, they risk compromising patients' understanding of their condition, their response to treatment, and their overall adherence. Nonadherence may increase when prescribers fail to establish a close relationship with their patients [13,114,132].

A deficient patient-provider relationship can be mitigated by employing a patient-centered approach [13,133,134]. Barriers related to healthcare that affect adherence include difficulty accessing care, formulary restrictions, formulary shifting, and the high costs of prescription drugs and copayments [16-18].

Adherence is also impacted by poor access to healthcare, such as living in areas with limited access, lacking transportation, having inadequate insurance, facing financial difficulties, and lacking other resources [135]. Long clinic wait times, long waiting periods for specialist appointments, and unclear healthcare referral processes are additional healthcare-related factors that may affect adherence [136,137].

Insurance plays a significant role in nonadherence. Problems include difficulties finding in-network healthcare providers, prescription drug plans that do not cover certain medications, and high copayments [13,138]. For instance, if a drug is not included in a patient’s restricted network formulary, physicians may be less likely to prescribe it. These restrictions affect the healthcare system, providers, and patients alike [139].

Polypharmacy, using multiple medications, can complicate treatment outcomes and adherence, especially regarding dosage, frequency, and application site [7,52,140]. Complex dermatological regimens often require the frequent use of several topical medications. Patients may become confused about which medications to use for specific conditions and how often to apply them. For example, treating scalp psoriasis often requires several medications (i.e., keratolytic agents and TCS), but patients may become less motivated to follow complex instructions [141].

**Figure 2:**
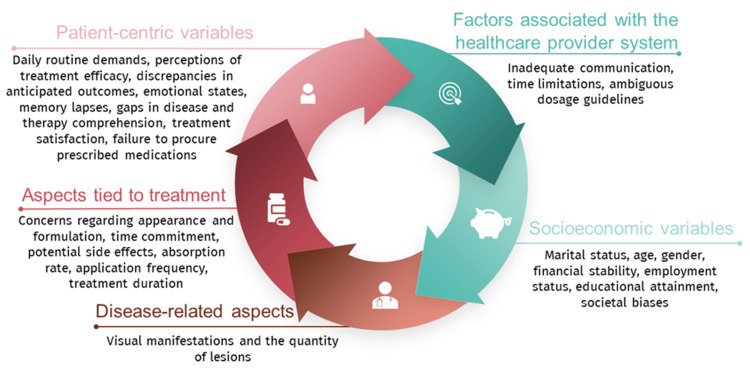
Categorization of the most important variables affecting the rate of adherence. Credits: Andrei Flavius Radu.

Barriers related to the patient, prescriber, or healthcare system can be classified as either intentional or unintentional reasons for nonadherence. Providers can help prevent unfilled prescriptions, maintain therapy, and improve clinical outcomes by identifying and addressing these barriers [13]. Pharmacists play a critical role in managing polypharmacy effectively, significantly contributing to its successful management [142]. Figure 2 summarizes the most relevant factors that affect adherence rates [143].

Measurement of treatment adherence

Adherence is typically assessed over a specified timeframe and expressed as a percentage, offering insights into dosage compliance compared to prescribed regimens. In some instances, adherence is categorized as a binary outcome (adherent/non-adherent), stratified into different levels (low/high adherers), or occasionally presented as an average value [144].

Various tools and metrics have been devised and validated to assess adherence and persistence in diverse diseases accurately and efficiently. Each method presents distinct advantages and drawbacks that necessitate careful consideration when selecting an appropriate approach. Direct assessment methods involve measuring drug or metabolite levels, offering precise and objective confirmation of drug consumption. However, these methods often come with elevated costs, invasiveness, and susceptibility to inter-individual variations [145,146]. Conversely, indirect evaluation techniques, such as pill counts, electronic databases, and self-reported measures, offer simplicity and cost-effectiveness. Nevertheless, they may lack direct evidence of drug ingestion, potentially overestimate adherence, and be vulnerable to recall bias. While direct methods afford meticulous evaluation, indirect approaches offer convenience and affordability, though they may compromise precision [145,147].

The role of the pharmacist in improving care in dermatological diseases

Promoting treatment adherence is an essential function of healthcare providers, including pharmacists [148]. If patients have questions or concerns about administering their medication, pharmacists are well-equipped with the knowledge and training to help them understand and follow their treatment plan [149].

A professional survey was completed by 456 pharmacists. A total of 64% had attended a dermatological course in college, and 38% had pursued further studies with a master's degree or higher. In the spring and summer, pharmacists reported sunburn and acne cases at 22.6% and 15.6%, respectively, while in the fall and winter, skin dryness at 18.8% and head lice at 13.1% were the most often reported conditions. Additionally, over 50% of the pharmacists reported that skincare recommendations typically accounted for 6%-15% of all recommendations. Regarding counseling patients with dermatological issues, 64% were either very confident or confident. Of all the reasons for consulting dermatologists, pharmacists cited "unsure of diagnosis" as the most prevalent. Pharmacists specializing in dermatology reported higher self-assurance in treating skin conditions.

Based on the findings, community pharmacists observe relatively few dermatologic illnesses on a regular basis, but most are confident in their ability to counsel and manage patients. However, they acknowledged a gap in dermatology training during their undergraduate and graduate studies [150].

Researchers interviewed pharmacists and parents of children (0-12 years old) with AD, both in person and over the phone. The interviews were recorded, transcribed verbatim, and analyzed using qualitative data analysis software. Themes were identified, including key performance indicators and perspectives on treatment from both pharmacy workers and parents. A total of 29 parents and 18 pharmacy employees participated. Intentional nonadherence due to fear of steroid side effects and issues with topical therapy administration were common concerns raised by many parents. Pharmacy workers reported similar issues, with some employees voicing concerns about steroid use. While most parents found the information helpful, some expressed a need for more specific recommendations on topics like clothing and bathing. Pharmacists and technicians frequently mentioned the inability to adequately advise patients on proper medication use as a common concern [151].

All Swedish pharmacies participated in a pharmaceutical care initiative known as "Skin Year" in 1993. The goal was to promote better treatment for skin diseases among pharmacy patients by raising awareness, improving adherence, and ensuring appropriate treatment. Pharmacies and other healthcare providers were the primary venues for campaign activities. The initiative provided pharmacies with promotional materials, and while the campaign's main target was pharmacy patients, it also included local healthcare professionals. Analyzing the campaign's impact on medication use in four disease categories, acne, dry skin, athlete's foot, and eczema, allowed for indirect evaluation without the need for expensive, time-consuming nationwide surveys. Medications used to treat skin conditions saw significant growth during 1993 and 1994, indicating increased awareness, adherence, and treatment appropriateness as mentioned in the campaign objectives [152].

Professional caregivers' skepticism about corticosteroids and inconsistent information delivery may amplify fears of using them among patients and parents of children with AD. A nationwide survey in France was conducted to gauge pharmacists' skepticism regarding topical steroid use for children with AD. A standardized 50-item questionnaire was sent to 500 randomly selected pharmacies (about 2% of the total) to assess their knowledge, trust, beliefs, and practices concerning topical steroid administration. Pharmacists rated their confidence in using topical steroids on a visual analogue scale from 0 to 10, with an average confidence score of 4.46. The study concluded that pharmacists' moderate confidence in topical steroids may affect future adherence rates when these drugs are prescribed [153].

Corticophobia, the fear of using corticosteroids, is a significant obstacle to treatment adherence. A study using the Topical Corticosteroid Phobia (TOPICOP) questionnaire evaluated healthcare professionals' levels of corticophobia. A professional version of the questionnaire (TOPICOP-P) was developed for dermatologists, general practitioners (GPs), pediatricians, and pharmacists. The overall TOPICOP score averaged 41.9 ± 14.9%. Pharmacists scored 48.5 ± 13.9%, GPs scored 46.0 ± 13.5%, pediatricians scored 39.7 ± 14.5%, and dermatologists scored 32.3 ± 12.1%. The mean scores among the four groups were significantly different. The study highlighted a significant fear of corticosteroids, particularly among GPs and pharmacists, and recommended re-education for healthcare providers to improve patient adherence [154].

**Figure 3:**
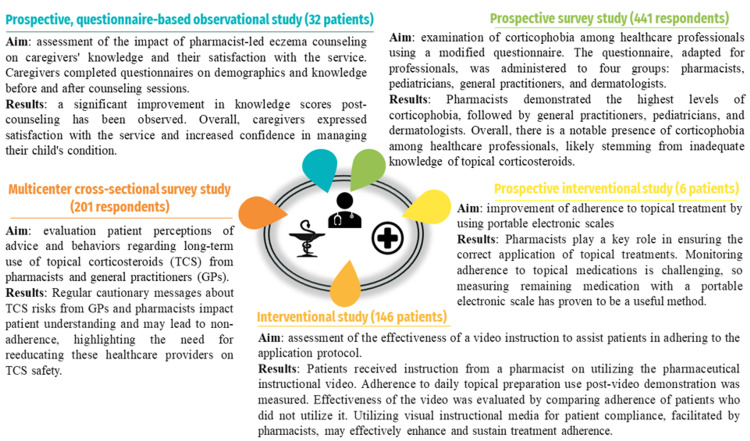
Synopsis of chosen clinical investigations evaluating pharmacist interventions for oral and/or topical treatment. Credits: Andrei Flavius Radu

Figure 3 provides an overview of relevant clinical studies evaluating the oral and/or topical therapeutic interventions provided by pharmacists [154-158].

Educating pharmacists and related specialties, and then counseling parents, has been suggested by some investigators as a solution. Researchers used a pre-post approach with a 3-month intervention duration to examine the efficacy of the intervention in pharmacies. A questionnaire was completed by parents and pharmacy personnel at baseline and during three-month follow-up appointments. Key performance indicators, anxieties, and opinions related to corticophobia were assessed using the TOPICOP questionnaire. The more negative the attitude, the higher the score. Nineteen pharmacy staff members and forty-eight parents who participated in a counseling session at the pharmacy provided both baseline and follow-up data. Negative beliefs and concerns about TCS decreased in both groups. When comparing parents and pharmacy employees, the mean total TOPICOP ratings dropped from 42% to 35% for parents and from 33% to 25% for pharmacy employees. These findings suggest that many parents exhibited corticophobia. Targeted patient counseling and the education of pharmacy staff appear to be effective in reducing corticophobia [159].

In another survey conducted after three months, patients were randomly distributed into two groups: one that received isotretinoin education from a clinical pharmacist alongside the doctor (treatment group), and another that received standard education from the doctor (control group). Using a validated questionnaire, patients' knowledge of isotretinoin’s optimal use, side effects, and management of those effects were assessed at baseline and three months after treatment. A total of 203 participants (103 from the treatment group and 100 from the control group) completed the trial. The intervention group outperformed the control group in terms of knowledge gained between baseline and follow-up scores.

Dermatology practices that offer clinical pharmacy services have the potential to lessen the impact of isotretinoin’s side effects by raising patients’ awareness of the drug, thus improving acne outcomes and enhancing patients' QoL [160].

An investigational study was conducted to evaluate the recommendations and actions of GPs and pharmacists regarding long-term TCS use, as reported by patients and parents of patients utilizing these medications. The international cross-sectional study included patients and parents who had used TCS for at least one month. Participants were asked about their treatment adherence, reasons for non-adherence, beliefs about TCS use and safety, and the information they received from GPs, pharmacists, family, friends, and the Internet. A total of 201 individuals participated, including 123 patients and 78 parents. Of 192 responses, 76.6% reported receiving messages about the risks of TCS from either their GP or pharmacist. A higher percentage of respondents reported that pharmacists, rather than GPs, recommended trying natural or alternative therapies before resorting to TCS [158].

There is great potential for pharmacists to help patients with dermatological diseases better adhere to their prescription regimens since pharmacists are significant and easily accessible healthcare providers. They manage treatment regimens, educate patients, and facilitate access to medications. Pharmacists frequently respond to patient inquiries about disease management and recommend over-the-counter medications. While improvements are needed in dermatological medication monitoring, education, and addressing corticophobia, pharmacists already have the tools to improve dermatological treatment. Future educational initiatives, such as dermatology certification programs offered by the Board of Pharmacy Specialists, could enhance pharmacists' understanding and confidence in treating dermatological conditions [22].

Innovations in technology that support adherence

Extensive research and advancements have been made in using various health information systems to improve adherence [161]. Dermatology, in particular, has shown a strong interest in exploring the potential of technology to enhance patient adherence due to its elevated rates of non-adherence [162,163]. Technological advancements, including electronic medical records, online patient-provider communication, and e-prescriptions, have become essential components of modern medical practice. In this era, technology plays a crucial role in the daily operations of healthcare. However, the concept of using technology to improve patient adherence is relatively recent [164]. Innovators, entrepreneurs, and healthcare professionals are rapidly entering this expanding field. The technologies involved can include physical devices, smartphones, telecommunications, and internet networks [165,166].

Table 1 presents the various technologies currently used or with potential applications for improving treatment adherence in patients with dermatological conditions.

**Table 1:** Advancements in technology aimed at fostering adherence.

Reference	Technology	Advantages	Effect on adherence
Okwundu N et al. (2018) [167] Alpalhão M et al. (2018) [168]	Mobile phone calls	Simplification and ease of access	Enhanced by phone call reminders and motivational calls
Svendsen MT et al. (2018) [169]	Smartphone applications	Accessibility and attention-grabbing features facilitated by the ability to set reminders using standard features	Increased due to the added benefit of providing patients with medication consumption data and allowing the measurement of psoriasis severity through symptom and photo diaries via a dedicated application
Boker A et al. (2012) [170]	Text reminders	Accessibility, Automated messaging tailored to medication administration methods	Increased through customized twice-daily text message reminders for applying acne medication
Tuong W and Armstrong AW (2015) [171] Tuong W et al. (2015) [172]	Web-Based Patient Education	Accessibility, User-friendly interface	Enhanced by educational videos, text-based content, and graphics detailing side effects, leading to a significant increase in adherence
Ferrándiz L et al. (2018) [173]	Teledermatology	Accessibility and cost-effectiveness	Despite the benefits they offer, teledermatology approaches exhibit a decline compared to face-to-face interactions, with the failure to initiate therapy being a significant factor contributing to treatment failure in teledermatology patients
Yentzer BA et al. (2011) [174]	Internet-Based Surveys	Accessibility and cost-effectiveness	Boosted by weekly email surveys assessing adherence, treatment regimen ease, efficacy, and experienced side effects
Lacour JP et al. (2017) [175]	Physical Devices	Assistance, reminders, and administration facilitation	Supported by the use of autoinjector devices, resulting in heightened patient satisfaction and confidence
Reich K et al. (2017) [62]	Multimodal approaches	Synergistic approaches	Enhanced through comprehensive guidance for communication between healthcare professionals and patients, alongside patient information materials and treatment reminders
Höchsmann C et al. (2019) [176]	Gamification*	Accessibility, Smartphone synchronization	Facilitated by compatibility with smartphones, resulting in increased intrinsic motivation for physical activity
Hoffmann C et al. (2018) [177]	Automated Medication Dispensers*	Digital pill box solutions and automation for memory facilitation	Strengthened by automated home medication dispensers, providing audio and visual reminders
Babel A et al. (2021) [178]	Artificial intelligence*	Potential for large-scale data assistance and evaluation	Enabled by managing reminders and contacting patients, with the potential to improve adherence

Many of these technologies are more feasible to integrate as they incorporate familiar technologies such as the internet, smartphone apps, and pre-existing smartphone reminder features. Additional advancements might involve the development of new tools (e.g., at-home phototherapy) or innovative uses of traditional instruments (e.g., gamification). It is important for the field of dermatology to observe how far technology has progressed to support adherence. However, regardless of the intervention, it is critical to recognize that adherence is inherently complex and that behavioral, psychological, and educational factors are essential [179].

## Conclusions

In dermatology therapy, nonadherence remains a common problem linked to poor clinical outcomes, unsuccessful treatments, and decreased QoL. While nonadherence is seen across all medical disciplines, the variety of treatment options available in dermatology warrants special attention. The duration of therapeutic management, the complexity of the regimen, and access to care are all important determinants of adherence. Simplifying treatment plans or increasing the frequency of office visits are two examples of interventions that can improve adherence. Accurately diagnosing and treating patients is crucial, but understanding and enhancing adherence is equally important due to its significant impact on costs, healthcare outcomes, and mortality.

Enhancing the patient-doctor relationship and establishing accountability are key to improving adherence. Without these foundations, other strategies are unlikely to be very successful. Once this groundwork is in place, targeted strategies can be employed to address common causes of nonadherence. Medication reminders and education can help overcome issues related to treatment inefficacy, beliefs, and forgetfulness. Physicians can use various strategies to improve treatment outcomes and adherence, including more advanced psychological measures such as minimizing exaggerated concerns about side effects, presenting the effectiveness of treatment in a more positive light, or leveraging technological advancements.
